# Characterization of Models for Identifying Physical and Cognitive Frailty in Older Adults With Diabetes: Systematic Review and Meta-Analysis

**DOI:** 10.2196/84617

**Published:** 2026-01-29

**Authors:** Xia Wang, Shujie Meng, Xiang Xiao, Liu Lu, Hongyan Chen, Yong Li, Rong Zhang, Qiwu Jiang, Shan Liu, Ru Gao

**Affiliations:** 1 School of Basic Medical Sciences and School of Nursing, Chengdu University, No. 2025, Chengluo Avenue, Chengdu, China; 2 School of Nursing, Yibin Vocational College of Medicine and Health, Yibin, China; 3 Nursing Department, The Fourth People's Hospital of Yibin, Yibin, China; 4 Rehabilitation College, Sichuan Health Rehabilitation Vocational College, Zigong, China; 5 School of Rehabilitation and Wellness, Yibin Vocational College of Medicine and Health, Yibin, China; 6 Nursing Department, Wenjiang District People's Hospital, No.86 Kangtai Road Wenjiang District, Chengdu, Sichuan Province, 611130, China, 86 18328690226

**Keywords:** prediction model, diabetes, frailty, systematic review, meta-analysis

## Abstract

**Background:**

Physical frailty and cognitive frailty are increasingly recognized as critical geriatric syndromes among older adults with diabetes, contributing to adverse outcomes such as disability, hospitalization, and mortality. Early identification of individuals at high risk is therefore essential for timely prevention and intervention. Although a growing number of prediction models have been developed for this population, evidence regarding their methodological rigor, predictive performance, and generalizability remains fragmented.

**Objective:**

This study aims to evaluate and characterize existing models for detecting or predicting physical frailty and cognitive frailty in older adults with diabetes.

**Methods:**

PubMed, Embase, Web of Science, China National Knowledge Infrastructure (CNKI), Wanfang, and VIP databases were searched from their inception to December 2025. Retrospective, cross-sectional, and prospective studies that developed or validated models predicting frailty or cognitive frailty in older adults with diabetes were included. The Prediction Model Study Risk Of Bias Assessment Tool (PROBAST) was used to assess risk of bias and applicability. Random effects meta-analyses using the Hartung-Knapp-Sidik-Jonkman method were conducted to synthesize model performance, including the pooled area under the receiver operating characteristic curve (AUC). Heterogeneity was explored through subgroup and sensitivity analyses. Small study effects were evaluated using funnel plots, the Egger test, and the Deeks funnel plot asymmetry test.

**Results:**

A total of 24 studies comprising 32 diagnostic models were included. The overall pooled analysis demonstrated an AUC of 0.851 (95% CI 0.820‐0.882) with a 95% prediction interval of 0.710‐0.992, sensitivity of 0.810 (95% CI 0.740‐0.850), and specificity of 0.850 (95% CI 0.810‐0.890). Statistical comparisons in the modeling approach revealed that logistic regression models achieved a significantly higher pooled AUC (0.850) compared with machine learning models (0.785; *P*=.003). Similarly, retrospective studies demonstrated superior performance, with an AUC of 0.900 compared with 0.843 for cross-sectional studies (*P*=.03). Conversely, no significant differences were observed across subgroups stratified by data source (*P*=.42), patient characteristics (*P*=.77), validation methods (*P*=.16), or specific outcomes (*P*=.94). The most common predictors identified were depression, age, and regular exercise; however, all included studies were assessed as having a high risk of bias.

**Conclusions:**

To our knowledge, this review provides the first comprehensive synthesis of models for risk stratification of physical frailty and cognitive frailty in older adults with diabetes. The findings indicate that existing models demonstrate satisfactory discrimination; specifically, CIs confirmed a robust average effect, while prediction intervals suggested that performance in future settings, though variable, is likely to remain acceptable. However, clinical utility is currently constrained by high risk of bias and limited external validation. Future research must prioritize rigorous, prospective, multicenter studies adhering to standard reporting guidelines (eg, TRIPOD [Transparent Reporting of a Multivariable Prediction Model for Individual Prognosis or Diagnosis]) to establish valid, generalizable, and clinically actionable prognostic instruments.

## Introduction

### Background

Diabetes mellitus has evolved into one of the most critical global public health challenges of the 21st century. According to the International Diabetes Federation (IDF), approximately 589 million adults were living with diabetes globally in 2024, with projections indicating this number could rise to 853 million by 2050 [1]. In the absence of optimal management, patients with diabetes are predisposed to micro- and macrovascular complications that significantly shorten life expectancy [2,3]. Recent data from the Global Burden of Disease study indicate that the prevalence of diabetes increases with age, reaching 24.4% among individuals aged ≥75 years [4]. Older adults are especially vulnerable to diabetes-related complications due to greater medical complexity and a higher likelihood of frailty compared with younger populations [5].

Frailty is regarded as a consequence of the decline in function and reserve of multiple organs with age, particularly involving the neuromuscular, endocrine, and immune systems [6]. Notably, frailty is particularly prevalent among patients with diabetes, with reported prevalence rates ranging from 10.4% to 20.8% across different studies [7-10]. Furthermore, previous studies indicated that individuals with diabetes have an approximately 1.6-fold higher risk of developing frailty than those without diabetes [11]. However, frailty frequently co-occurs with cognitive impairment [12]; their simultaneous presence is termed cognitive frailty, a distinct clinical entity that represents a crucial subtype of frailty requiring specific attention.

Once established, frailty typically follows a progressive trajectory, increasing the likelihood of adverse clinical outcomes such as falls, incontinence, rapid functional decline, pressure ulcers, and delirium [13-17]. In addition to these risks, frailty is linked to higher rates of hospitalization, emergency department visits, prolonged inpatient stays, and mortality [18,19]. Of particular concern is that the coexistence of physical and cognitive impairment further amplifies these risks, leading to greater adverse outcomes [20,21].

Evidence suggests a bidirectional relationship between diabetes and frailty, often creating a cycle where each condition exacerbates the other [22]. The presence of physical or cognitive frailty introduces significant complexity to diabetes management [23]. In frail patients, physiological deterioration and multi-organ dysfunction fundamentally alter the pharmacokinetics of antihyperglycemic agents [24]. Specifically, sarcopenia, increased adiposity, and compromised renal or hepatic clearance heighten the susceptibility to adverse drug events, such as hypoglycemia and unintended weight loss. Additionally, the decreased caloric intake typical of this population further aggravates the risk of hypoglycemia and hinders recovery from hypoglycemic events [25,26].

In recent years, physical frailty and cognitive frailty have been increasingly conceptualized as dynamic and potentially preventable or reversible conditions, especially when identified at an early stage [27,28]. In patients with diabetes, nonpharmacological interventions—including structured physical activity, nutritional optimization, and multimodal strategies—have demonstrated potential benefits for mitigating frailty progression. Consequently, early identification of individuals at high risk has become a cornerstone of effective prevention and management strategies. To this end, diagnostic and prognostic models designed to detect physical or cognitive frailty integrate multiple demographic, clinical, and psychosocial factors to estimate an individual’s risk profile. These models serve to support health care professionals with stratifying risk, facilitating timely and targeted interventions, and optimizing the allocation of health care resources.

However, the clinical application of these models may be hindered due to insufficient evidence regarding their performance, risk of bias, and applicability in routine practice. Although individual studies exist, no systematic review has yet comprehensively evaluated these models for both physical frailty and cognitive frailty in older adults with diabetes. Therefore, it is essential to conduct a systematic review that thoroughly assesses the methodological quality and clinical applicability of existing models.

### Objectives

The aim of this systematic review and meta-analysis was to evaluate the methodological quality and clinical utility of existing models designed for the identification or prediction of physical frailty and cognitive frailty in older adults with diabetes. The specific aims included the following: (1) to determine the characteristics and most frequent predictors of risk prediction models developed for physical frailty and cognitive frailty in this population; (2) to analyze the methodological limitations and risk of bias of these models using the Prediction Model Study Risk Of Bias Assessment Tool (PROBAST); and (3) to investigate the pooled predictive performance of these tools to assess their potential for real-world clinical implementation.

## Methods

### Search Strategy and Selection Criteria

This systematic review and meta-analysis was registered on PROSPERO (CRD420251019308). The study followed the PRISMA (Preferred Reporting Items for Systematic Reviews and Meta-Analyses) expanded checklist [29] and the PRISMA extension for diagnostic test accuracy (PRISMA-DTA) [30], while the literature search was conducted and reported in accordance with PRISMA-S (PRISMA Search) [31]. A comprehensive literature search was conducted across the PubMed, EMBASE, Web of Science, China National Knowledge Infrastructure (CNKI), Wanfang, and VIP databases, covering records from database inception to December 2025. We developed the search strategy based on the PITROS (Participants, Index Test, Target Conditions, Reference Standard, Outcomes, Settings) framework (Table S1 in Multimedia Appendix 1). The strategy combined Medical Subject Headings (MeSH) with free-text terms (Table S2 in Multimedia Appendix 1). The search strategy was independently evaluated by another librarian in accordance with the PRESS (Peer Review of Electronic Search Strategies) guidelines. In addition, references of relevant studies, guidelines, and reviews were manually searched, and citation tracking was performed using the Web of Science database to identify other relevant studies. No study registries were searched.

In clinical settings, prediction encompasses both diagnostic models (estimating the probability of a particular condition being present) and prognostic models (forecasting the likelihood of future outcomes) [32,33]. This review included all primary studies describing the development and/or validation of prediction models, tools, or scores for estimating the risk of physical frailty or cognitive frailty in older adults with diabetes. The inclusion criteria were (1) participant age ≥60 years and presence of diabetes, including diabetes only and diabetes with other comorbidities or complications; (2) research content involving the construction of a predictive model for identifying physical frailty or cognitive frailty in individuals with diabetes; (3) retrospective studies, cross-sectional studies, and prospective studies; and (4) published in English or Chinese. The exclusion criteria were (1) duplicate publications; (2) reviews, case reports, or conference abstracts; (3) literature that could not be obtained from the original text; (4) literature that could not provide valid data; and (5) studies in which the model contained only a single predictor.

### Data Extraction

This study used the reference management software EndNote X9 to identify and remove duplicate records. We then eliminated literature unrelated to the research topic by screening titles and abstracts. Finally, the full texts were reviewed to identify studies satisfying both the inclusion and exclusion criteria. Upon completion of the literature screening process, a data extraction form was devised in accordance with the Checklist for Critical Appraisal and Data Extraction for Systematic Reviews of Prediction Modeling Studies (CHARMS) [34]. The contents included (1) basic information of the study including author, year of publication, study location, study design, and source of data used; (2) patient characteristics including sample size and patient diagnosis; (3) number of predictors, predictor type, most important predictors, and predictor screening methods; (4) model characteristics including outcome indicators, modeling methods, model verification methods, and missing data processing methods; (5) model presentation; and (6) model performance as measured using the area under the curve (AUC), sensitivity, and specificity. In studies where multiple models were developed and the best-performing model was explicitly reported, we included the best model in the analysis. For studies that reported multiple models without specifying a preferred one, we selected the model with the highest AUC to represent the study. We prioritized internally validated estimates, resorting to development performance or external validation data only when internal estimates were unavailable. For studies with unclear or incomplete data, attempts were made to contact the corresponding authors. To ensure the consistency and accuracy of the final data, two researchers (XW and SM) independently extracted the data, and the extracted results were compared and checked. Inconsistencies were resolved through discussion and consultation, and a third researcher (RG) was asked to assist in judgment when necessary.

### Quality Assessment

Two reviewers independently used PROBAST [35] to appraise the risk of bias and the applicability of each included study. Disagreements were resolved by discussion or by consulting a third reviewer. PROBAST evaluates 4 domains: participants, predictors, outcome, and analysis. Each domain is rated as high, unclear, or low risk of bias. The applicability evaluation focuses on the 3 areas of research (subjects, predictors, and results), and its evaluation process is similar to the risk of bias assessment.

### Statistical Analysis

Meta-analyses were conducted using Stata 18 (specifically the midas and metan commands) and R version 4.3.2 (the metafor package). Using AUC values derived from models, we calculated the pooled AUC and produced an AUC forest plot. An AUC below 0.7 signified inadequate discrimination, an AUC ranging from 0.7 to 0.8 denoted moderate discrimination, and an AUC exceeding 0.8 suggested excellent discrimination [36]. Additionally, models that reported sample sizes and sensitivity and specificity were extracted. The true positive (TP), false positive (FP), false negative (FN), and true negative (TN) for each model were calculated using the formulas sensitivity=TP/(TP+ FN) and specificity=TN/(FP+ TN). Pooled sensitivity and pooled specificity were computed based on TP, FP, FN, and TN, and corresponding forest plots of sensitivity and specificity were constructed. Subsequently, a summary receiver operating characteristic curve was generated. The degree of heterogeneity across the models under consideration was evaluated using the *Q* test and measured using the *I*² statistic (where an *I*² value <25% signifies low heterogeneity, between 25% and 50% indicates moderate heterogeneity, and >50% denotes high heterogeneity) [37]. To account for between-study heterogeneity and provide more robust variance estimation, we used the Hartung-Knapp-Sidik-Jonkman method for a random effects meta-analysis on the logit scale [38]. For studies with significant heterogeneity, subgroup analyses, sensitivity analyses, or only descriptive analyses were performed. The presence of small study effects was evaluated using the Egger test, funnel plots, and the Deeks funnel plot [39]. A *P* value <.05 was deemed to indicate statistical significance.

## Results

### Study Selection

**Figure 1.**
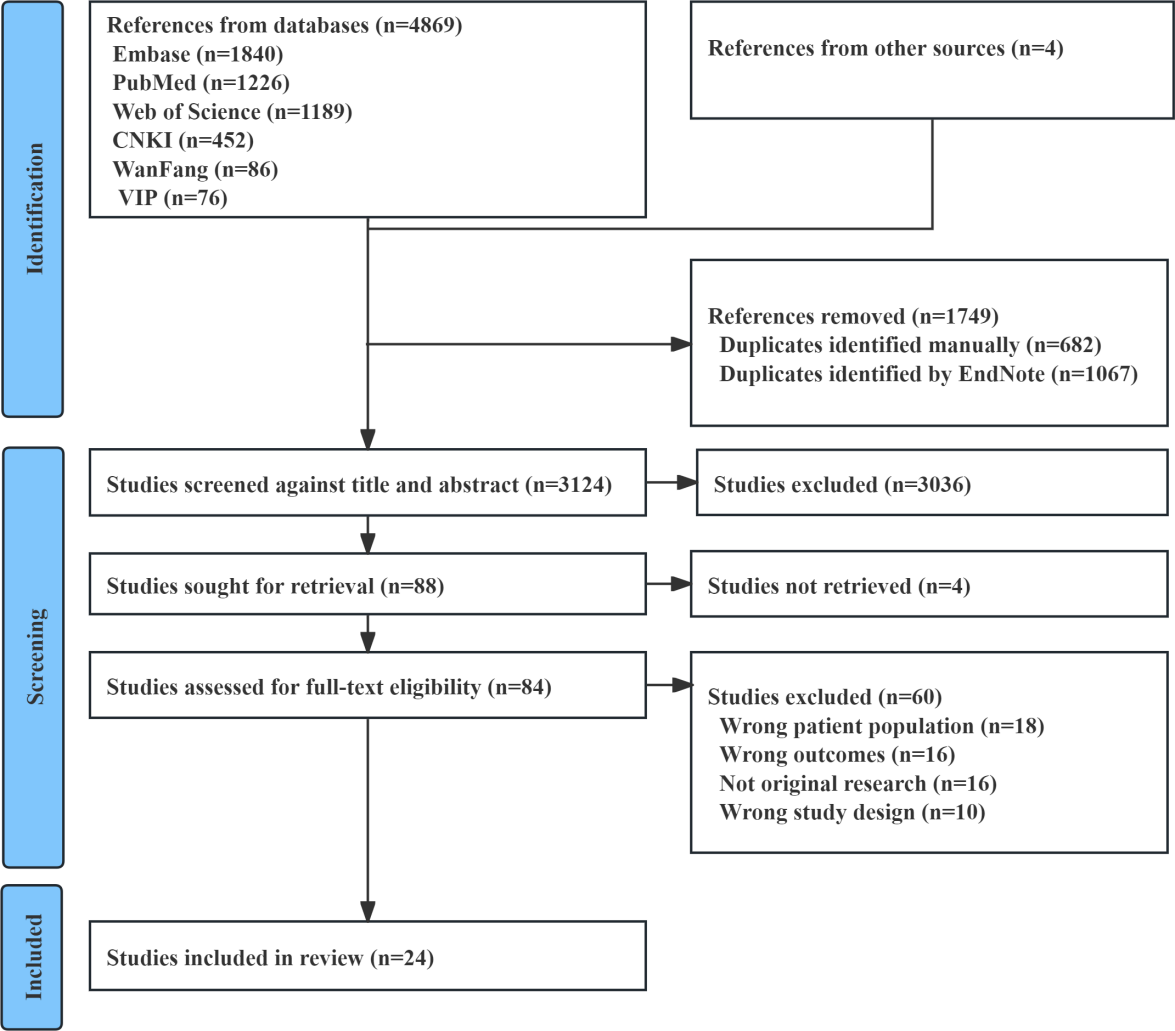
Literature screening flow chart. CNKI: China National Knowledge Infrastructure.

The initial search identified a total of 4873 records. After removing duplicates, 3124 records remained. Following title and abstract screening, 88 studies were selected for full-text review. We could not retrieve 4 studies. During the full-text assessment, 18 studies were excluded because the study population did not have diabetes mellitus. Additionally, 16 studies were excluded because the outcome variable was not physical frailty or cognitive frailty; 16 were excluded because they were nonoriginal studies, such as reviews and meta-analyses; and 10 were excluded due to an inappropriate study design. Ultimately, 24 studies [40-63] were included in this meta-analysis. The literature screening process and results are illustrated in Figure 1.

### Characteristics of Included Studies

The specific characteristics of the included studies are detailed in Tables1  2. A total of 24 studies reported 32 diagnostic models for physical frailty and cognitive frailty in older adults with diabetes. The publication years of these papers spanned from 2023 to 2025. All the studies were conducted in China. The number of participants in the included studies ranged from 152 to 1436. The prevalence of frailty varied from 10.1% to 51.2%, while that of cognitive frailty ranged from 20.3% to 62.1%. Regarding study design, 21 studies [41-46,48-60,62,63] used a cross-sectional design, while 3 were retrospective studies [40,47,61]. In addition, 18 studies [41-48,50,51,53-59,63] were conducted at single centers, whereas 6 studies [40,49,52,60-62] were multicenter studies. In terms of the target population, 7 studies [40-42,57,58,60,61] included all types of diabetes, 14 studies [43,45,47-51,53-56,59,62,63] focused specifically on patients with type 2 diabetes, and 3 studies [44,46,52] included patients with diabetes and other comorbidities or complications. Physical frailty was the primary outcome in 13 studies [40-42,44,45,51,52,55,57,58,60-62], while the remaining 11 studies [43,46-50,53,54,56,59,63] investigated cognitive frailty. For frailty screening, 22 studies [40-51,53-57,59-63] used the Fatigue, Resistance, Ambulation, Illnesses, and Loss of Weight (FRAIL) scale, while 2 studies [52,58] used the Tilburg Frailty Indicator. To evaluate cognitive function in the 11 cognitive frailty studies, the Montreal Cognitive Assessment was the predominant tool used in 10 studies [43,46-50,53,54,59,63], whereas the Mini-Mental State Examination was used in only 1 study [56]. Regarding data handling, missing data were not reported in 9 studies [42,43,45-47,49,51,56,59], 3 studies [40,57,62] used imputation methods, and 12 studies [41,44,48,50,52-55,58,60,61,63] excluded participants with missing data. Continuous variables were maintained as continuous in 14 studies [40,42-44,47,51,52,55-59,61,62] and transformed into categorical variables in 10 studies [41,45,46,48-50,53,54,60,63]. The majority of models were presented as nomograms: Specifically, 13 studies [40,43,45,48,50,51,54-57,59,61,63] presented results solely as nomograms, 4 studies [41,42,44,46] provided both nomograms and full equations, 1 study [52] presented a nomogram and risk chart, and 1 study [58] presented a nomogram and decision tree. Additionally, 3 studies [53,60,62] developed risk sum scores, and 2 studies [47,49] provided logistic regression (LR) equations.

**Table 1.** Overview of the basic characteristics of included studies (n=24) identifying physical and cognitive frailty in older adults with diabetes.

Author	Year	Outcome	Definition of outcome	Sample size, n	Event rate, n (%)	Modeling algorithms used	Population	Internal validation	Predictors, n	Model presentation
Wu et al [62]	2025	Frailty	FRAIL^a^	509	148 (29.1)	LR^b^, SVM^c^, GBM^d^, RF^e^, CatBoost	T2DM^f^	Cross-validation	7	Sum score
Xiao et al [61]	2025	Frailty	FRAIL	1107	113 (10.2)	LR	Diabetes	Random split	7	Nomogram
Wang et al [55]	2025	Frailty	FRAIL	152	47 (31. 1)	LR	T2DM	Random split	4	Nomogram
Du et al [45]	2024	Frailty	FRAIL	458	83 (18.1)	LR	T2DM	Bootstrap	8	Nomogram
Tang et al [51]	2024	Frailty	FRAIL	566	213 (37.6)	LR	T2DM	Random split	6	Nomogram
Wang et al [52]	2024	Frailty	TFI^g^	491	216 (44.0)	LR, NN^h^	Diabetes with diabetic foot	Random split	8	Nomogram and risk chart
Dang [42]	2024	Frailty	FRAIL	360	115 (32.0)	LR	Diabetes	Random split (+ external)	5	Nomogram and full equation
Xi [57]	2024	Frailty	FRAIL	338	130 (38.5)	LR	Diabetes	Random split	6	Nomogram
Cheng [41]	2024	Frailty	FRAIL	317	118 (37.2)	LR	Diabetes	Bootstrap	5	Nomogram and full equation
Yin [58]	2024	Frailty	TFI	379	194 (51.2)	LR, DT^i^	Diabetes	Bootstrap	8	Nomogram and decision tree
Zheng [60]	2024	Frailty	FRAIL	380	112 (29.5)	RF, SVM, KNN^j^	Diabetes	Cross-validation	7	Sum score
Bu et al [40]	2023	Frailty	FRAIL	1436	145 (10.1)	LR	Diabetes	Random split	7	Nomogram
Dong et al [44]	2023	Frailty	FRAIL	485	211 (43.5)	LR	Diabetes with diabetic retinopathy	Bootstrap	7	Nomogram and full equation
Ma et al [49]	2025	Cognitive frailty	FRAIL, MoCA^k^	253	76 (30.0)	LR	T2DM	None	5	Full equation
Wang et al [53]	2025	Cognitive frailty	FRAIL, MoCA	202	80 (39.6)	DT	T2DM	Random split	11	Sum score
Liang et al [46]	2024	Cognitive frailty	FRAIL, MoCA	265	93 (35.1)	LR	Diabetes with COPD^l^	Random split (+ external)	7	Nomogram and full equation
Yu and Yu [63]	2024	Cognitive frailty	FRAIL, MoCA	430	132 (30.7)	LR	T2DM	Bootstrap (+ external)	7	Nomogram
Zhang et al [59]	2024	Cognitive frailty	FRAIL, MoCA	215	66 (30.7)	LR	T2DM	Bootstrap	5	Nomogram
Liu [47]	2024	Cognitive frailty	FRAIL, MoCA	220	137 (62.1)	LR	T2DM	Random split	4	Full equation
Deng et al [43]	2023	Cognitive frailty	FRAIL, MoCA	315	87 (27.6)	LR	T2DM	Random split	6	Nomogram
Wang and Xu [54]	2023	Cognitive frailty	FRAIL, MoCA	262	85 (32.4)	LR	T2DM	Bootstrap	8	Nomogram
Wang et al [56]	2023	Cognitive frailty	FRAIL, MMSE^m^	321	85 (26.5)	LR	T2DM	Bootstrap	5	Nomogram
Liu [48]	2023	Cognitive frailty	FRAIL, MoCA	483	98 (20.3)	LR	T2DM	Random split	6	Nomogram
Meng [50]	2022	Cognitive frailty	FRAIL, MoCA	508	117 (23.0)	LR	T2DM	Bootstrap (+ external)	6	Nomogram

a FRAIL: Fatigue, Resistance, Ambulation, Illness, and Loss of Weight scale.

b LR: logistic regression.

c SVM: support vector machine.

d GBM: gradient boosting machine.

e RF: random forest.

f T2DM: type 2 diabetes mellitus.

g TFI: Tilburg Frailty Indicator

h NN: neural network.

i DT: decision tree.

j KNN: k-nearest neighbors.

k MoCA: Montreal Cognitive Assessment.

l COPD: chronic obstructive pulmonary disease.

m MMSE: Mini-Mental State Examination.

**Table 2.** Methodological and clinical characteristics of included studies (n=24) identifying physical frailty and cognitive frailty in older adults with diabetes.

Characteristic	Studies, n (%)
Study design	
Retrospective studies	3 (13)
Cross-sectional study	21 (88)
Source of data used	
Single center	18 (75)
Multicenter	6 (25)
Missing data handling	
Not reported	9 (38)
Exclusion	12 (50)
Imputation	3 (13)
Handling of continuous data	
Continuous	14 (58)
Categorical or dichotomous	10 (42)
Feature selection	
Univariate analysis	6 (25)
Multivariate analysis	7 (29)
Univariate analysis and multivariate analysis	11 (46)
Calibration method	
Hosmer-Lemeshow test	2 (8)
Calibration plot	4 (17)
Hosmer-Lemeshow test and calibration plot	14 (58)
None	4 (17)
Validation method	
Internal validation	19 (79)
External validation and internal validation	4 (17)
None	1 (4)

### Characteristics of Included Prediction Models

Regarding modeling methods, among the 32 models included, LR was the most commonly used algorithm. LR analyses were used in 22 models, while 10 models used machine learning (ML) techniques, including random forest (n=2), support vector machines (n=2), decision trees (n=2), k-nearest neighbors (n=1), CatBoost (n=1), gradient boosting machine (n=1), and neural networks (n=1). Model discrimination was reported for all models, with AUC values ranging from 0.703 to 0.983 (Table S3 in Multimedia Appendix 1). Specificity and sensitivity were reported in 17 studies [40-42,44,46-50,52,53,56-58,60-62] involving 25 models. Specifically, sensitivity ranged from 0.102 to 0.955, and specificity varied from 0.505 to 0.990. However, model calibration was not reported in 4 studies [52,53,60,62]. *P* values from both the Hosmer-Lemeshow test and calibration plots were used in 14 studies [40-44,48,50,51,54-59], 2 studies [47,49] used *P* values only from the Hosmer-Lemeshow test, and 4 studies [45,46,61,63] used only calibration plots. Regarding model validation, 1 study [49] developed models without validation, and 19 studies [40,41,43-45,47,48,51-62] conducted only internal validation without external validation.

### Features

**Figure 2.**
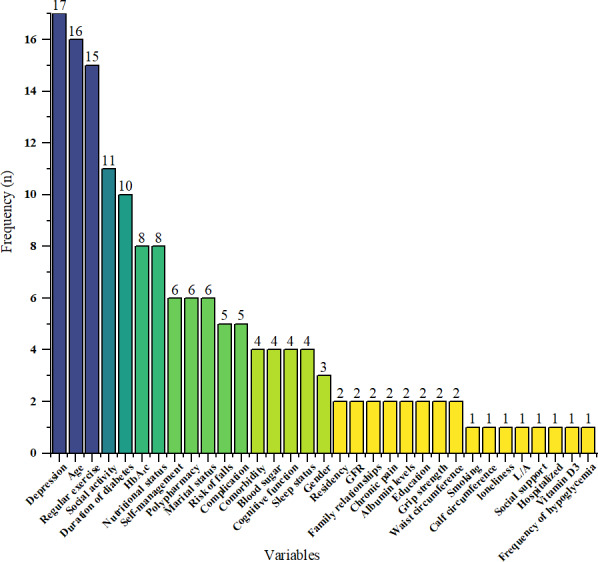
Frequency of predictors in the included studies. GFR: glomerular filtration rate; HbA_1c_: glycated hemoglobin; L/A: ratio of serum leptin to adiponectin.

All features covered a wide range of factors, including sociodemographic characteristics, lifestyle factors, health-related factors, mental health status, laboratory test indicators, and anthropometric measurements. A total of 33 features were involved in the studies. The number of features incorporated into each study varied from 4 to 11. Among the features, the 5 most frequently occurring were depression, age, regular exercise, social activity, and duration of diabetes. The frequency distribution of all features is illustrated in Figure 2.

### Quality Assessment

#### Overview

**Figure 3.**
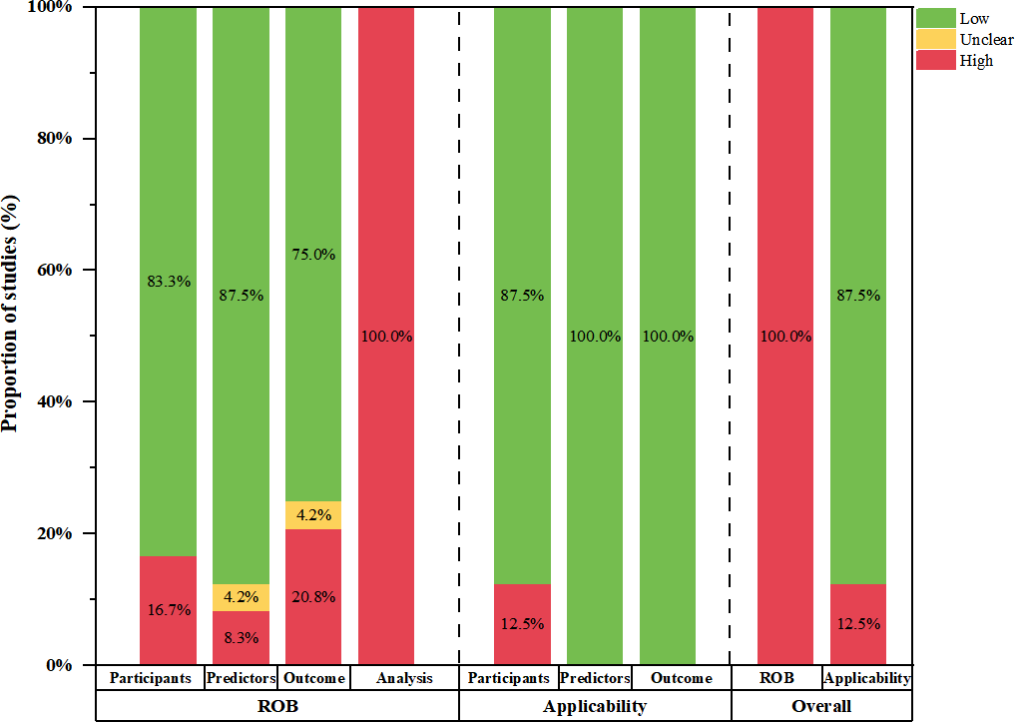
Prediction Model Study Risk Of Bias Assessment Tool (PROBAST) risk of bias (ROB) and applicability assessment for all included studies.

The PROBAST tool was used to assess the risk of bias and the applicability of the included prediction model studies (Figure 3 and Table S4 in Multimedia Appendix 1). According to the established criteria, all 24 studies, which encompassed 32 models, were identified as having a high risk of bias. In terms of applicability, 13 studies [43,44,46-50,52-54,56,59,63], including 14 models, were deemed to have high concerns regarding applicability. Conversely, the remaining 11 studies [40-42,45,51,55,57,58,60-62], which included 18 models, were considered to have low concerns regarding applicability. Notably, 4 studies [52,58,60,62] included multiple models each; however, there was no difference in the quality assessment results between the models within these studies.

#### Risk of Bias Assessment

Within the participant domain, 4 studies [40,43,47,61] were recognized as exhibiting a high risk of bias. Of these, 3 studies [40,47,61] were deemed as having a high risk owing to their study designs, while the remaining study [43] was classified as such due to the exclusion of specific subgroups that could potentially alter the performance of the prediction model. In the predictor domain, 2 studies [40,63] were assessed as having a significant risk of bias due to the use of outcome information in the evaluation of predictors, 1 study [55] was rated as having an unclear risk of bias because the researchers did not report whether they used the same assessment measures when evaluating the predictors. In the outcome domain, 3 studies [40,62,63] had a significant risk of bias because the definition of outcomes included ≥1 predictor, 2 studies [52,53] were rated as having a high risk of bias due to the potentially inappropriate time interval between predictor assessment and outcome determination, and 1 study [49] was deemed to be at unclear risk of bias as they did not report information on the method of outcome classification. In the analysis domain, all studies were judged to have a high risk of bias. Current guidance recommends that studies developing predictive models achieve at least 20 events per variable (EPV). However, 13 studies [43,45,46,48,49,53-56,59-61,63] did not meet this requirement. Moreover, 10 studies [41,45,46,48-50,53,54,60,63] transformed continuous variables into categorical variables, either in part or entirely, and the authors did not report whether standard definitions were used for the categorization; 1 study [40] partially excluded participants for unreasonable reasons. Regarding the handling of missing data, 12 studies [41,42,44,48,50,52-54,58,60,61,63] directly excluded cases with missing data, while 9 studies [43,45-47,49,51,55,56,59] did not explicitly report whether data were missing. In addition, 6 studies [44,49,50,52,54,59] did not avoid selecting variables based solely on univariate analysis; 3 studies [52,53,60] did not comprehensively assess the predictive performance of their models, using only discrimination measures without calibration; 6 studies [41,44,48,50,55,57] neglected to evaluate the risk of overfitting, underfitting, or optimism that could bias the apparent performance of their predictive models; 1 study [49] developed models without validation; and 19 studies [40,41,43-45,47,48,51-62] conducted only internal validation without external validation.

#### Applicability Risk Assessment

In the participant domain, 3 studies [44,46,52] had a high risk of applicability concerns due to the inclusion of individuals with other comorbidities or complications.

### Meta-Analysis

**Figure 4.**
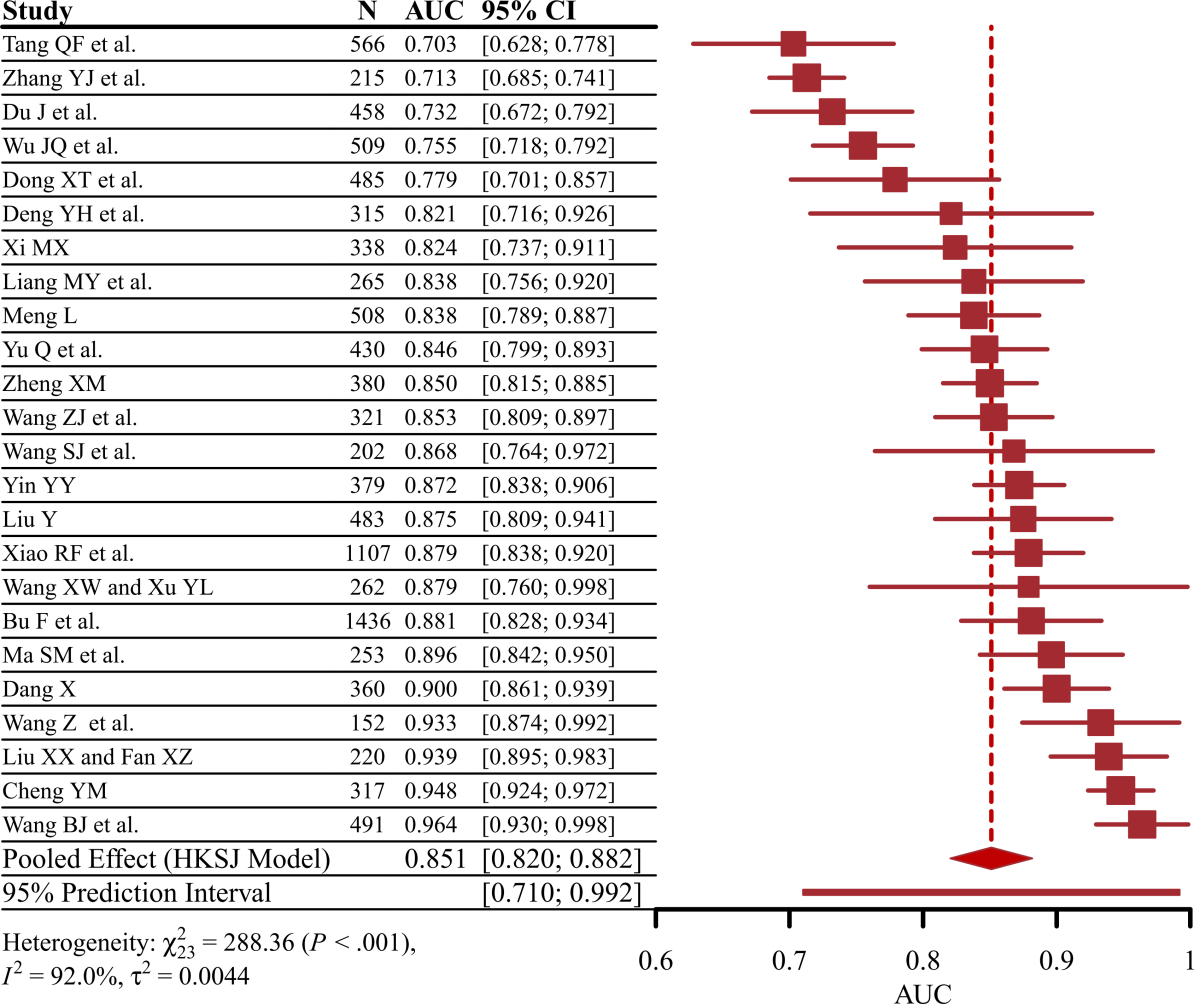
Forest plot of the random effects meta-analysis of pooled area under the curve (AUC) estimates for 29 validation models [40-62]. HKSJ: Hartung-Knapp-Sidik-Jonkman.

A random effects meta-analysis using the Hartung-Knapp-Sidik-Jonkman method was performed to evaluate the predictive performance at both the study and model levels. Regarding the analysis of the 24 included studies, the overall pooled AUC was 0.851 (95% CI 0.820‐0.882), with a 95% prediction interval (PI) of 0.710 to 0.992 (*P*<.001; *I*²=92.0%; Figure 4). When analyzing the 32 models, the overall pooled AUC was 0.829 (95% CI 0.802‐0.856), with a 95% PI of 0.686 to 0.972 (*P*<.001; *I*²=92.5%; Figure S1 in Multimedia Appendix 1).

**Figure 5.**
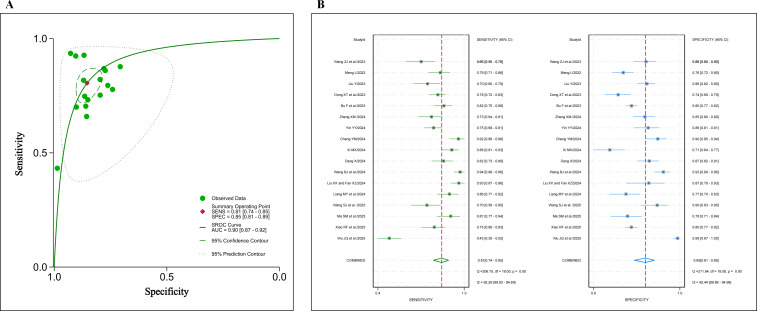
(A) Summary receiver operating characteristic curve and (B) forest plots of the random effects meta-analysis of the sensitivity and specificity for 22 validation models [40-42,44,46-50,52,53,56-58,60-62]. AUC: area under the curve; SENS: sensitivity; SPEC: specificity; SROC: summary receiver operating characteristic.

Additional data on sample size, sensitivity, and specificity were extracted from 17 studies to calculate TP, FP, FN, and TN (Table S5 in Multimedia Appendix 1). Based on these values, the pooled sensitivity was 0.810 (95% CI 0.740‐0.850; *I*²=92.26%), as illustrated in the forest plot. The pooled specificity was 0.850 (95% CI 0.810‐0.890; *I*²=92.44%), with the corresponding forest plot also presented (Figure 5B). Furthermore, a summary receiver operating characteristic curve was generated, as depicted in Figure 5A. These results indicate significant heterogeneity across the models regarding AUC, sensitivity, and specificity.

### Sensitivity Analysis

**Figure 6.**
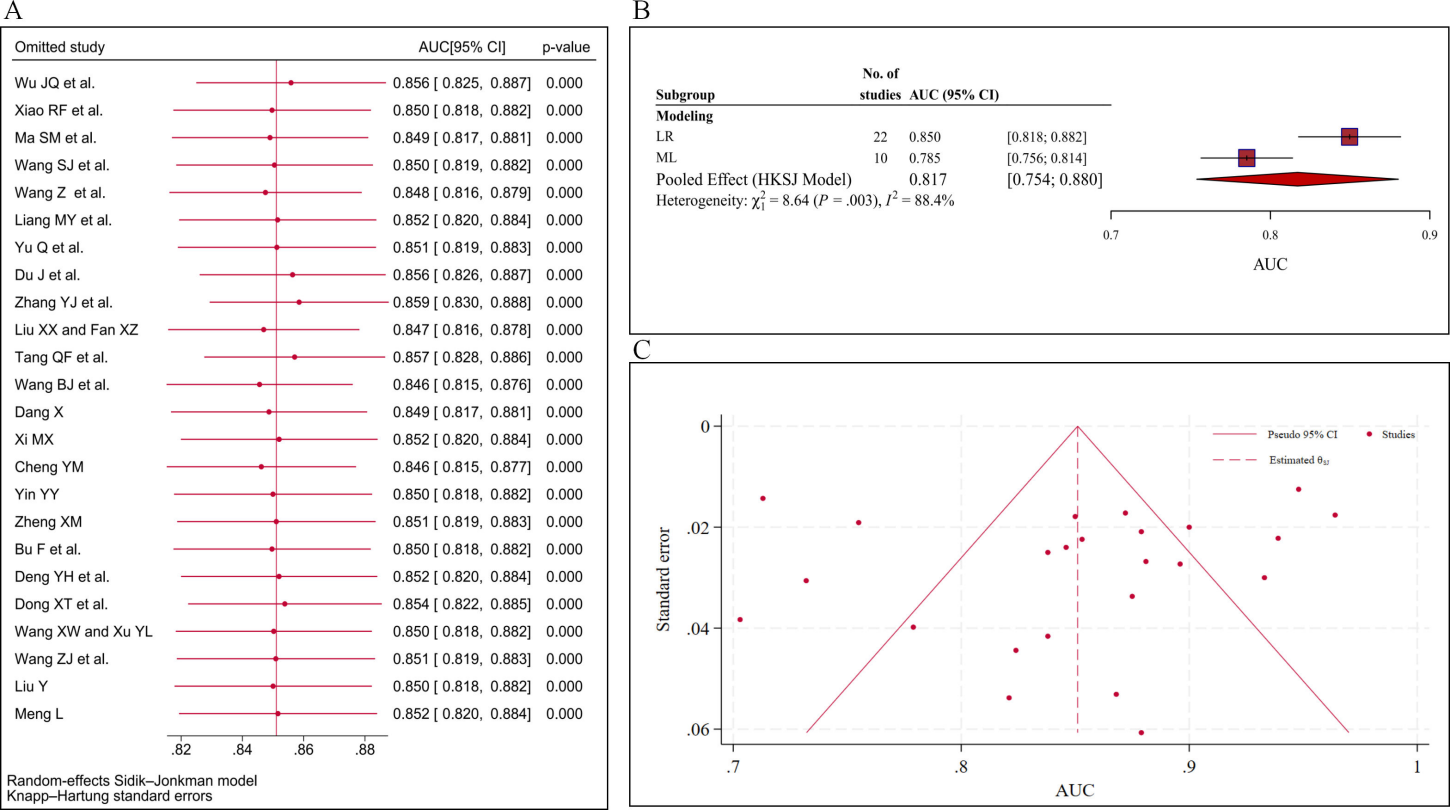
Assessment of robustness, heterogeneity, and small study effects in the meta-analysis of 24 studies evaluating physical frailty and cognitive frailty in older adults with diabetes: (A) sensitivity analysis, (B) subgroup forest plot stratified by modeling approach (32 models), and (C) funnel plot [40-63]. AUC: area under the curve; HKSJ: Hartung-Knapp-Sidik-Jonkman; LR: logistic regression; ML: machine learning.

Sensitivity analyses were undertaken by sequentially removing one study at a time. The point estimates obtained after excluding any single study all fell within the 95% CI of the overall effect size (Figure 6A). This indicates that the removal of any individual study did not significantly influence the pooled AUC. Therefore, the combined results were relatively stable.

### Subgroup Analysis

Subgroup analyses were performed based on modeling approach, study design, data source, population, outcome, and validation method (Table 3 and Figure 6B). Significant differences were observed in modeling approaches (*P*=.003), where LR models yielded a higher pooled AUC (0.850) compared with ML models (0.785). Similarly, study design showed significant heterogeneity (*P*=.03), with retrospective studies demonstrating superior diagnostic performance (AUC=0.900) than cross-sectional studies (AUC=0.843). However, no statistically significant differences were found across subgroups stratified by data source (*P*=.42), patient characteristics (*P*=.77), validation methods (*P*=.16), or specific outcomes (*P*=.94).

**Table 3.** Subgroup analysis of the pooled area under the curve (AUC) of studies of physical frailty and cognitive frailty in older adults with diabetes to explore potential sources of heterogeneity (n=24).

Subgroup	Studies, n	AUC (95% CI)	*I*^2^, %	*P* value
Population			0	.77
Diabetes	21	0.849 (0.818-0.879)		
Diabetes with comorbidities or complications	3	0.866 (0.757-0.975)		
Study design			79.6	.03
Cross-sectional study	21	0.843 (0.812-0.875)		
Retrospective study	3	0.900 (0.861-0.939)		
Data source			0	.42
Multicenter	6	0.871 (0.815-0.926)		
Single center	18	0.844 (0.809-0.878)		
Outcome			0	.94
Physical frailty	13	0.851 (0.806-0.896)		
Cognitive frailty	11	0.849 (0.812-0.886)		
Validation			42.5	.16
Random split	12	0.875 (0.836-0.914)		
Bootstrap	9	0.829 (0.779-0.879)		
Cross-validation	2	0.803 (0.710-0.896)		
None	1	0.896 (0.842-0.950)		

### Small Study Effects Assessment

We examined small study effects using the Egger test, funnel plots, and the Deeks funnel plot. The Egger test yielded a coefficient of −1.07 (*P*=.40), indicating no statistically significant small study effects. This result aligns with the visual inspection of the contour-enhanced funnel plot (Figure 6C), which displayed a relatively symmetrical distribution of the studies, suggesting no obvious small study effects among the included studies. Furthermore, the Deeks funnel plot asymmetry test yielded a nonsignificant *P* value of .94, providing no evidence of significant small study effects (Figure S2 in Multimedia Appendix 1).

However, these results must be interpreted with caution. Funnel plot symmetry or nonsignificant statistical tests do not definitively rule out publication bias. Conversely, asymmetry may arise from heterogeneity or methodological limitations not merely from publication bias. Therefore, the potential for publication bias cannot be entirely excluded, particularly due to limitations in study selection. Although our search strategy was designed to be global, the final pool of eligible studies consisted exclusively of research conducted in China. Additionally, the restriction of inclusion criteria to English and Chinese languages may have introduced language bias by excluding relevant data published in other languages.

## Discussion

### Principal Findings

To our knowledge, this is the first systematic review and meta-analysis to comprehensively evaluate the performance of prediction models for physical frailty and cognitive frailty specifically in older adults with diabetes. Our analysis included 24 studies encompassing 32 models. At the study level, the pooled AUC was 0.851 (95% CI 0.820‐0.882), while the model-level analysis yielded a similarly high pooled AUC of 0.829 (95% CI 0.802‐0.856). In addition, the pooled sensitivity was 0.810, and the pooled specificity was 0.850, indicating that these models demonstrated reasonable discriminative performance for identifying physical frailty and cognitive frailty in this high-risk population.

A notable finding from our subgroup analysis was the difference in performance based on the modeling approach (*P*=.003). LR models yielded a higher pooled AUC (0.850) than ML models (0.785). However, this finding should be interpreted with caution. Consistent with the systematic review by Christodoulou et al [64], we observed no consistent performance advantage of ML over LR analysis in this dataset. The apparent disparity may be attributed to the smaller number of ML studies included, heterogeneity in populations and predictors, and potential risk of bias, rather than an inherent superiority of LR. For instance, Wang et al [52] and Yin [58] developed models using both approaches and found LR performed slightly better. Ultimately, there is no “one-size-fits-all” modeling method; performance often depends on the specific data structure and clinical context. Therefore, future research should prioritize rigorous comparisons of multiple modeling approaches—including proper hyperparameter tuning for ML—to identify the optimal strategy for specific physical frailty and cognitive frailty prediction scenarios. Additionally, we observed that retrospective studies yielded significantly higher AUC values (0.900) than cross-sectional studies (0.843; *P*=.03). This phenomenon likely stems from the inherent selection bias and better data quality control often present in retrospective cohorts, potentially leading to overoptimistic performance estimates.

The diagnostic models included in this review possess meaningful clinical implications, facilitating a shift toward the precise identification and risk stratification of physical frailty and cognitive frailty in clinical settings. Our analysis revealed that the most frequently used features—depression, age, regular exercise, social activity, and diabetes duration—provide concrete metrics for identifying these concurrent conditions. Notably, depression emerged as the most consistent and prominent feature across multiple studies, highlighting its strong correlation with both physical frailty and cognitive frailty. Existing evidence indicates a bidirectional relationship between depression and these conditions, potentially mediated through inflammatory pathways, endocrine dysregulation, and overlapping symptoms such as fatigue and psychomotor slowing [65,66]. Consequently, assessing mental health not only is vital for psychological well-being but also serves as a critical entry point for identifying patients who may already be experiencing physical frailty or cognitive frailty. Advanced age was also a predominant feature, consistent with reports by Kong et al [9] and Wang et al [67]. This association likely reflects immunosenescence, chronic inflammation, and metabolic dysregulation, which collectively contribute to sarcopenia and functional decline [68-70]. These findings suggest that age remains a fundamental stratification factor, warranting heightened clinical vigilance for physical and cognitive frailty in older cohorts. Regular exercise was identified as a powerful discriminatory feature. Physiologically, physical activity is known to reduce inflammation, preserve muscle function, and support cognitive health [71]. In the context of these diagnostic models, the absence of regular exercise serves as a robust, easily accessible clinical marker for detecting potential physical frailty and cognitive frailty. This allows health care providers to efficiently target vulnerable populations in community settings where elaborate geriatric assessments may be impractical. Similarly, lower levels of social activity emerged as a significant indicator. This suggests that social isolation often co-occurs with physical frailty and cognitive frailty, making social history a valuable component of the risk stratification process for older adults with diabetes. Finally, the duration of diabetes was a frequent feature in the included models. The strong association between longer disease duration and physical frailty and cognitive frailty likely reflects the cumulative burden of chronic hyperglycemia, insulin resistance, and related complications over time [8,26]. As diabetes duration increases, physiological and cognitive reserves decline, thereby increasing the probability of concurrent physical frailty and cognitive frailty. These insights emphasize that patients with a long history of diabetes represent a high-risk group requiring prioritized screening and comprehensive management.

### Comparison With Prior Work

Previous systematic reviews and narrative summaries on physical frailty or cognitive frailty in older adults with diabetes have primarily concentrated on estimating prevalence, identifying associated risk factors, or describing frailty phenotypes, rather than systematically evaluating multivariable prediction or identification models [8,9,72,73]. Moreover, most prior reviews did not attempt a quantitative synthesis of model performance, likely due to the substantial methodological and clinical heterogeneity across studies, which is commonly encountered in the evaluation of prediction models. Consistent with this literature, we observed considerable variation among included studies in terms of study populations, definitions of physical frailty and cognitive frailty, predictor selection, model development strategies, and validation approaches. These differences reflect the evolving and fragmented nature of model development in this field and pose challenges for direct comparison across studies. Nevertheless, by conducting a meta-analysis of discrimination performance, particularly through the synthesis of the AUC, our study provides a quantitative overview of the overall performance of existing models for identifying physical frailty and cognitive frailty in older adults with diabetes.

### Heterogeneity

Substantial heterogeneity was observed across the included studies (*I*²>90% for sensitivity, specificity, and AUC). This is a common challenge in diagnostic meta-analyses and may be attributed to variations in study design, population characteristics, and modeling methodologies. Our subgroup analysis identified that the modeling approach (ML vs LR) and study design (retrospective vs cross-sectional) were significant sources of heterogeneity (*P*<.05). However, other factors such as data source (single vs multicenter) and outcome definitions (physical frailty vs cognitive frailty) did not significantly contribute to the observed variance. It is also important to note the variability in feature selection across studies. With 33 different features identified—ranging from depression and age to regular exercise—the lack of a standardized set of predictors likely contributes to the heterogeneity in model performance. This diversity reflects the multifaceted pathophysiology of frailty in diabetes but complicates the direct comparison of models.

Importantly, beyond the conventional *I*² statistic, we further quantified between-study heterogeneity using 95% PIs, which provide a clinically meaningful estimate of the expected range of model performance in future settings. Although *I*² values exceeding 90% indicate substantial relative heterogeneity, they do not convey the absolute extent to which predictive performance may vary across populations and clinical contexts. In contrast, PIs directly address this limitation by reflecting the dispersion of true effects on the original AUC scale. At the study level, the pooled AUC of 0.851 was accompanied by a 95% PI ranging from 0.710 to 0.992, indicating that, although predictive performance may vary considerably across different real-world settings, most future applications are still likely to achieve at least acceptable discrimination.

### Methodological Quality and Risk of Bias

Evaluation using the PROBAST checklist indicated that all included studies exhibited a high risk of bias, predominantly in the analysis domain. Consequently, the pooled performance estimates reported in this review should be interpreted with caution, likely representing “best-case scenarios” or optimistic estimates rather than robust predictions of real-world performance.

In the participant domain, specifically regarding data sources, although retrospective designs were identified as a source of bias for a few studies, the overarching issue remains the analytical approach. We recommend using prospective data or registry data for model development in future optimization efforts to reduce the risk of bias arising from data sources. Additionally, the evaluation of model applicability indicated that certain studies included not only patients with diabetes but also those with other comorbidities or complications. These factors limited the applicability of the respective models to the general diabetes population. In the outcome domain, some studies inappropriately incorporated outcome-related information into the predictor assessment, leading to information leakage and inflated model performance. In addition, inadequate reporting regarding the consistency of predictor measurement raised concerns about reproducibility in at least one study. In the outcome domain, several studies were judged to have a high risk of bias due to problematic outcome definitions.

The analysis domain had the highest frequency of a high risk of bias, with all studies rated as high risk in this domain. According to the PROBAST assessment tool, an EPV≥20 is commonly used as a heuristic to indicate an adequate sample size for developing prediction models. In this review, 13 studies had an EPV <20, which may suggest an increased risk of bias related to model overfitting. However, relying solely on fixed rules of thumb may be insufficient; therefore, future studies should prioritize formal, model-tailored sample size calculations (eg, approaches proposed by Riley et al [74]) to ensure precise estimation and adequate statistical power. During the predictor selection process, several studies relied solely on univariate screening, which often fails to identify confounding factors and can lead to model overfitting. Therefore, predictor selection should not solely depend on univariate screening but should also be combined with clinical practice. Moreover, an increasing number of studies are using least absolute shrinkage and selection operator (LASSO) regression to handle high-dimensional data and select potential variables. By introducing a penalty term, LASSO regression reduces the estimates of extreme variables, thereby effectively enhancing the accuracy of model estimation and decreasing the likelihood of overfitting [75]. Moreover, the handling of missing data was suboptimal. Cases with missing data were directly excluded in 12 studies, which can introduce bias and reduce statistical power, while 9 studies failed to report how missing data were handled. A minority of studies used appropriate methods such as multiple imputation. Accurate data reporting and careful handling of missing observations help reduce model overfitting. It is recommended that future studies strengthen the management of missing data to ensure the integrity of the study. When dealing with continuous variables, transforming continuous data into categorical variables for modeling may lead to a significant loss of model efficacy [76]; however, 10 studies in our review performed such transformations without reporting standard definitions. Although data transformation can be considered to enhance the convenience of application for researchers during the clinical dissemination phase, it should be done with caution during development. Crucially, the majority of models lacked external validation. Although they demonstrated good discrimination in derivation cohorts, their performance remains inherently tied to their specific development settings.

Therefore, the primary implication of this review is methodological: Rather than endorsing specific existing tools for immediate clinical use, we emphasize the urgent need for better-designed research. Future studies must strictly adhere to TRIPOD (Transparent Reporting of a Multivariable Prediction Model for Individual Prognosis or Diagnosis) guidelines and PROBAST standards—specifically ensuring adequate sample sizes, appropriate handling of continuous variables and missing data, and rigorous external validation in independent, geographically distinct populations—to develop robust and transportable prediction models.

### Limitations

This study has several limitations that warrant consideration. First, the predominance of cross-sectional and retrospective designs among the included studies restricts the scope of these models to the diagnostic identification of prevalent frailty. Consequently, they function as concurrent screening tools rather than prognostic instruments for predicting future incidence, precluding the ability to infer causal relationships between predictors and outcomes. Second, according to the PROBAST assessment, all included studies exhibited a high risk of bias, particularly within the analysis domain. This methodological weakness likely results in overoptimistic performance estimates and limits the transportability of these models to diverse clinical populations. Third, substantial statistical heterogeneity (*I*²) was observed, stemming from variations in study design and modeling methodologies. We calculated 95% PIs to provide a more clinically meaningful estimate of the expected range of model performance in future settings. Finally, all included studies were conducted in China, and the review was restricted to English and Chinese literature. Although this reflects the rapid emergence of this research focus within China, the lack of geographic and ethnic diversity limits the generalizability of our findings to other global populations and health care systems.

### Conclusion

This review provides the first comprehensive synthesis of models for risk stratification of physical and cognitive frailty in older adults with diabetes. The findings indicate that existing models demonstrate satisfactory pooled discriminative performance. Specifically, although the CIs confirm a robust average effect, the 95% PIs indicate that the distribution of predictive performance in future real-world settings is expected to vary across different clinical contexts, yet likely remaining within an acceptable range. Nevertheless, their clinical utility is currently constrained by significant methodological limitations. Specifically, the identified models rely heavily on readily available clinical and psychosocial predictors, such as depression, age, regular exercise, and social activity, suggesting that early risk stratification is feasible in routine practice. However, the evidence is underpinned by a pervasive high risk of bias, primarily due to analytical shortcomings, small sample sizes, and a lack of rigorous external validation. Furthermore, the predominance of cross-sectional designs and the geographic restriction of studies to China limit the generalizability of these tools to broader global populations and their ability to function as true prognostic instruments for future risk. Consequently, although current models show promise for screening and identifying prevalent physical and cognitive frailty, they are not yet sufficiently robust for widespread deployment in diverse clinical settings. Future research must pivot from developing new, redundant models to conducting robust, prospective, multicenter studies that adhere strictly to TRIPOD guidelines. Emphasis should be placed on external validation and the development of longitudinal prognostic tools to ensure reliable, transportable, and clinically actionable risk stratification for this vulnerable population.

## Supplementary material

10.2196/84617 Multimedia Appendix 1 Inclusion criteria, search terms, performance measures, and assessments of bias.

10.2196/84617 Checklist 1 PRISMA checklist.

## Abbreviations

AUC: area under the curve
CHARMS: Checklist for Critical Appraisal and Data Extraction for Systematic Reviews of Prediction Modeling Studies
CNKI: China National Knowledge Infrastructure
EPV: events per variable
FN: false negative
FP: false positive
FRAIL: Fatigue, Resistance, Ambulation, Illnesses, and Loss of Weight
IDF: International Diabetes Federation
LASSO: least absolute shrinkage and selection operator
LR: logistic regression
MeSH: Medical Subject Headings
ML: machine learning
PI: prediction interval
PITROS: Participants, Index Test, Target Conditions, Reference Standard, Outcomes, Settings
PRESS: Peer Review of Electronic Search Strategies
PRISMA: Preferred Reporting Items for Systematic Reviews and Meta-Analyses
PROBAST: Prediction Model Study Risk Of Bias Assessment Tool
TN: true negative
TP: true positive
TRIPOD: Transparent Reporting of a Multivariable Prediction Model for Individual Prognosis or Diagnosis
