# Correction: Characterization of Models for Identifying Physical and Cognitive Frailty in Older Adults With Diabetes: Systematic Review and Meta-Analysis

**DOI:** 10.2196/92717

**Published:** 2026-02-12

**Authors:** Xia Wang, Shujie Meng, Xiang Xiao, Liu Lu, Hongyan Chen, Yong Li, Rong Zhang, Qiwu Jiang, Shan Liu, Ru Gao

**Affiliations:** 1 School of Basic Medical Sciences and School of Nursing, Chengdu University, Chengdu, China; 2 School of Nursing, Yibin Vocational College of Medicine and Health, Yibin, China; 3 Nursing Department, The Fourth People's Hospital of Yibin, Yibin, China; 4 Rehabilitation College, Sichuan Health Rehabilitation Vocational College, Zigong, China; 5 School of Rehabilitation and Wellness, Yibin Vocational College of Medicine and Health, Yibin, China; 6 Nursing Department, Wenjiang District People's Hospital, No.86 Kangtai Road Wenjiang District, Chengdu, Sichuan Province, 611130, China, 86 18328690226

In “Characterization of Models for Identifying Physical and Cognitive Frailty in Older Adults With Diabetes: Systematic Review and Meta-Analysis” [1], the authors noted one correction.

Affiliation 5 has been changed from the following:

Medical and Nursing College, Yibin Vocational College of Medicine and Health, Yibin, China

The affiliation now reads:

School of Rehabilitation and Wellness, Yibin Vocational College of Medicine and Health, Yibin, China

The correction will appear in the online version of the paper on the JMIR Publications website, together with the publication of this correction notice. Because this was made after submission to PubMed, PubMed Central, and other full-text repositories, the corrected article has also been resubmitted to those repositories.
