# Supplementary material for: Characterization of Models for Identifying Physical and Cognitive Frailty in Older Adults With Diabetes: Systematic Review and Meta-Analysis
Source: J Med Internet Res. 2026 Jan 29;28:e84617. doi: 10.2196/84617 (PMC12854664; doi:10.2196/84617)

# Multimedia Appendix

# **Title: Characterization of Models for Identifying Physical and Cognitive Frailty in Older Adults with Diabetes: A Systematic Review and Meta-analysis**

# **Tables**

**Table S1.** Summary of inclusion criteria using the Participants, Index Test,

Target Conditions, Reference Standard, Outcomes, Settings (PITROS) framework.

| Item | Definition |
| --- | --- |
| Population(P) | Patients diagnosed with diabetes mellitus . |
| Index test (I) | Prediction models, risk scores, screening tools, or machine learning algorithms designed to identify or predict frailty or cognitive frailty. |
| Target conditions (T) | Frailty or cognitive frailty in patients with diabetes, defined according to established diagnostic or assessment criteria (e.g., frailty phenotype, frailty index, clinical frailty scale, or validated cognitive assessment tools). |
| Reference standard (R) | Established frailty or cognitive frailty assessment instruments used as reference standards in the primary studies, including validated frailty scales and cognitive assessment tools. |
| Outcomes (O) | Model performance measures, including discrimination (area under the curve [AUC] or C-statistic), and when available, calibration metrics, sensitivity, and specificity. |
| Settings (S) | Studies conducted in community, outpatient, or hospital settings using cross-sectional, retrospective, or prospective data sources. |

**Table S2.** Search strategy for identifying prediction and classification models of frailty and cognitive frailty in elderly patients with diabetes.

|  | Search | Query |
| --- | --- | --- |
| PubMed | #1 | ((((Diabetes Mellitus[MeSH Terms]) ) OR (Diabetes Complications[MeSH Terms])) OR (Diabetes Mellitus, Type 2[MeSH Terms])) OR (Diabetes Mellitus, Type 1[MeSH Terms]) |
|  | #2 | (((((((Diabetes Mellitus[Title/Abstract]) OR (Diabetes[Title/Abstract])) OR (type 2 diabetes[Title/Abstract])) OR (Diabetic[Title/Abstract])) OR (T2DM[Title/Abstract])) OR (T1DM[Title/Abstract])) OR (type 1 diabetes[Title/Abstract])) OR (Diabet*[Title/Abstract]) |
|  | #3 | (#1 )OR( #2) |
|  | #4 | (Frailty[MeSH Major Abstract]) |
|  | #5 | ((((((((Frailness[Title/Abstract]) OR (Debility[Title/Abstract])) OR (Debilities[Title/Abstract])) OR (Fatigue[Title/Abstract])) OR (Weakness[Title/Abstract])) OR (Frailty[Title/Abstract])) OR (Asthenia[Title/Abstract]) OR (Cognitive Frailty[Title/Abstract])) OR (Cognitive Impairment[Title/Abstract])OR (Frail*[Title/Abstract]) |
|  | #6 | (#4) OR( #5) |
|  | #7 | (((predictive value of tests[MeSH Major Abstract]) OR (nomogram[MeSH Major Abstract])) OR (prediction algorithms[MeSH Major Abstract])) OR (risk assessment[MeSH Major Abstract]) |
|  | #8 | ((((((((((((predictive value of tests[Title/Abstract]) OR (nomogram[Title/Abstract])) OR (Prediction Algorithms[Title/Abstract])) OR (risk assessment[Title/Abstract])) OR (predict*[Title/Abstract])) OR (predict* model[Title/Abstract])) OR (diagnostic model[Title/Abstract])) OR (forecasting[Title/Abstract])) OR (risk prediction[Title/Abstract])) OR (prognostic model[Title/Abstract])) OR (prediction tool[Title/Abstract])) OR (risk score[Title/Abstract])) OR (logistic model[Title/Abstract]) |
|  | #9 | (#7 )OR( #8) |
|  | #10 | ((#3) AND (#6)) AND (#9) |
| EMBASE | #1 | 'diabetes complications':ab,ti OR 'diabetes mellitus, type 2':ab,ti OR 'diabetes mellitus, type 1':ab,ti OR 'type 2 diabetes':ab,ti OR 'type 1 diabetes':ab,ti OR diabetes:ab,ti OR 'diabetes mellitus':ab,ti OR t2dm:ab,ti OR t1dm:ab,ti OR diabet*:ab,ti |
|  | #2 | frailty:ab,ti OR frailness:ab,ti OR debility:ab,ti OR debilities:ab,ti OR fatigue:ab,ti OR weakness:ab,ti OR frail*:ab,ti OR asthenia:ab,ti OR 'cognitive frailty':ab,ti OR 'cognitive impairment':ab,ti |
|  | #3 | predictive value of tests':ab,ti OR nomogram:ab,ti OR 'prediction algorithms':ab,ti OR 'risk assessment':ab,ti OR predict*:ab,ti OR 'predict* model':ab,ti OR prediction:ab,ti OR forecasting:ab,ti OR 'risk prediction':ab,ti OR 'prognostic model':ab,ti OR 'prediction tool':ab,ti OR 'risk score':ab,ti OR 'logistic model':ab,ti OR 'diagnostic model':ab,ti |
|  | #4 | #1 AND #2 AND #3 |
| Web of science | #1 | Diabetes Mellitus (Abstract) or Diabetes Complications (Abstract) or type 2 diabetes (Abstract) or Diabetic (Abstract) or T2DM (Abstract) or T1DM (Abstract) or type 1 diabetes (Abstract) or Diabet* (Abstract) |
|  | #2 | Frailty (Abstract) or Frailness (Abstract) or Debility (Abstract) or Debilities (Abstract) or Fatigue (Abstract) or Weakness (Abstract) or Asthenia (Abstract) or Cognitive Frailty (Abstract) or Cognitive Impairment (Abstract) or Frail* (Abstract) |
|  | #3 | predictive value of tests (Abstract) or nomogram (Abstract) or Prediction Algorithms (Abstract) or risk assessment (Abstract) or predict* (Abstract) or diagnostic model (Abstract) or forecasting (Abstract) or risk prediction (Abstract) or prognostic model (Abstract) or prediction tool (Abstract) or risk score (Abstract) or logistic mode (Abstract) |
|  | #4 | #1 AND #2 AND #3 |
| CNKI | #1 | (主题:糖尿病)OR(篇关摘:糖尿病(精确))OR(关摘:2型糖尿病(精确))OR(篇关摘:1型糖尿病(精确)) |
|  | #2 | (主题:衰弱)OR(篇关摘:衰弱(精确))OR(篇关摘:衰弱综合征(精确))OR(篇关摘:衰弱症(精确)) |
|  | #3 | (主题:列线图)OR(篇关摘:列线图(精确))OR(篇关摘:预测模型(精确))OR(篇关摘:预测(精确))OR(篇关摘:风险评估(精确))OR(篇关摘:诺莫图(精确))OR(篇关摘:风险预测(精确))OR(篇关摘:预警(精确))OR(篇关摘:模型(精确))OR(篇关摘:风险评分(精确)) |
|  | #4 | #1 AND #2 AND #3 |
| Wanfang | #1 | 主题:(糖尿病) or 题名或关键词:(糖尿病) or 题名或关键词:(1型糖尿病) or 题名或关键词:(2型糖尿病) |
|  | #2 | 主题:(衰弱) or 题名或关键词:(衰弱) or 题名或关键词:(衰弱综合征) or 题名或关键词:(衰弱症) |
|  | #3 | 主题:(列线图) or 题名或关键词:(列线图) or 题名或关键词:(预测模型) or 题名或关键词:(风险评估) or 题名或关键词:(诺莫图) or 题名或关键词:(风险预测)or [题名或关键词:(预警) or 题名或关键词:(模型) or 题名或关键词:(风险评分)](http://s-wanfangdata-com-cn-s.vpn.cdu.edu.cn:8118/advanced-search/paper?q=%E9%A2%98%E5%90%8D%E6%88%96%E5%85%B3%E9%94%AE%E8%AF%8D:(%E9%A2%84%E8%AD%A6) or %E9%A2%98%E5%90%8D%E6%88%96%E5%85%B3%E9%94%AE%E8%AF%8D:(%E6%A8%A1%E5%9E%8B) or %E9%A2%98%E5%90%8D%E6%88%96%E5%85%B3%E9%94%AE%E8%AF%8D:(%E9%A3%8E%E9%99%A9%E8%AF%84%E5%88%86)&searchtype=expert&type=["periodical","thesis"]&chineseEnglishExpand=true" \t "http://s-wanfangdata-com-cn-s.vpn.cdu.edu.cn:8118/advanced-search/_blank) |
|  | #4 | #1 AND #2 AND #3 |
| VIP | #1 | [((题名或关键词=糖尿病 OR 题名或关键词=1型糖尿病) OR 题名或关键词=2型糖尿病)](http://qikan-cqvip-com-s.vpn.cdu.edu.cn:8118/Qikan/search/index?LngMySearHistoryIdGuid=d470eb63-bd4e-4e58-885a-cf07fd62d0b7&from=Qikan_Article_History) |
|  | #2 | [((题名或关键词=衰弱 OR 题名或关键词=衰弱症) OR 题名或关键词=衰弱终综合症)](http://qikan-cqvip-com-s.vpn.cdu.edu.cn:8118/Qikan/search/index?LngMySearHistoryIdGuid=dac8d9c5-298f-4354-9715-365d0d6b9d44&from=Qikan_Article_History) |
|  | #3 | ([(((((题名或关键词=列线图 OR 题名或关键词=预测模型) OR 题名或关键词=风险评估) OR 题名或关键词=诺莫图) OR 题名或关键词=风险预测)](http://qikan-cqvip-com-s.vpn.cdu.edu.cn:8118/Qikan/search/index?LngMySearHistoryIdGuid=1ceb04fa-51d6-40c8-9957-0fb29907feb8&from=Qikan_Article_History) OR 题名或关键词=预警) OR 题名或关键词=风险评分) |
|  | #4 | #1 AND #2 AND #3 |

**Table S3.**  Discriminative performance of included 32 models for frailty and cognitive frailty in elderly patients with diabetes.

| Author | Modeling | Development performance | Internal validation | External validation |
| --- | --- | --- | --- | --- |
| Wu JQ et al. | LR^a^ | - | 0.741 | - |
| Wu JQ et al. | SVM^b^ | - | 0.740 | - |
| Wu JQ et al. | GBM^c^ | - | 0.748 | - |
| Wu JQ et al. | RF^d^ | - | 0.751 | - |
| Wu JQ et al. | CatBoost | - | 0.755 | - |
| Xiao RF et al. | LR | - | 0.879 | - |
| Ma SM et al. | LR | 0.896 | - | - |
| Wang SJ et al. | DT^e^ | 0.908 | 0.868 | - |
| Wang Z et al. | LR | 0.840 | 0.933 | - |
| Liang MY et al. | LR | - | - | 0.838 |
| Yu Q et al. | LR | - | 0.846 | 0.824 |
| Du J et al. | LR | 0.768 | 0.732 | - |
| Zhang YJ et al. | LR | 0.924 | 0.713 | - |
| Liu XX and Fang XZ | LR | 0.975 | 0.939 | - |
| Tang QF et al. | LR | 0.790 | 0.703 | - |
| Wang BJ et al. | LR | 0.973 | 0.964 | - |
| Wang BJ et al. | NN^f^ | 0.742 | 0.732 | - |
| Dang X | LR | - | 0.900 | 0.890 |
| Xi MX | LR | 0.851 | 0.824 | - |
| Cheng YM | LR | 0.969 | 0.948 | - |
| Yin YY | LR | 0.865 | 0.872 | - |
| Yin YY | DT | 0.783 | 0.813 | - |
| Zheng XM | RF | - | 0.850 | - |
| Zheng XM | SVM | - | 0.830 | - |
| Zheng XM | KNN^g^ | - | 0.790 | - |
| Bu F et al. | LR | 0.912 | 0.881 | - |
| Deng YH et al. | LR | 0.866 | 0.821 | - |
| Dong XT et al. | LR | 0.763 | 0.779 | - |
| Wang XW and Xu YL | LR | 0.897 | 0.879 | - |
| Wang ZJ et al. | LR | 0. 798 | 0.853 | - |
| Liu Y | LR | 0.886 | 0.875 | - |
| Meng L | LR | - | 0.838 | 0.895 |

^a^LR: logistic regression.

^b^SVM: support vector machine.

^c^GBM: gradient boosting machine.

^d^RF: random forest.

^e^DT: decision tree.

^f^NN: neural network.

^g^KNN: k-nearest neighbors.

-:indicates absence.

**Table S4.** Risk of bias and applicability assessment using the Prediction model Risk Of Bias Assessment Tool (PROBAST) for included studies to identify frailty and cognitive frailty in elderly patients with diabetes (N = 24).

| Author | No. of model | ROB^a^ | | | | Applicability | | | Overall | |
| --- | --- | --- | --- | --- | --- | --- | --- | --- | --- | --- |
|  |  | Participants | Predictors | Outcome | Analysis | Participants | Predictors | Outcome | ROB | Applicability |
| Ma SM et al. | 1 | +^b^ | + | ?^c^ | -^d^ | + | + | + | - | + |
| Wang SJ et al. | 1 | + | + | - | - | + | + | + | - | + |
| Wang Z et al. | 1 | + | ? | + | - | + | + | + | - | + |
| Liang MY et al. | 1 | + | + | + | - | - | + | + | - | - |
| Yu Q et al. | 1 | + | - | - | - | + | + | + | - | + |
| Du J et al. | 1 | + | + | + | - | + | + | + | - | + |
| Zhang YJ et al. | 1 | + | + | + | - | + | + | + | - | + |
| Liu XX and Fang XZ | 1 | - | + | + | - | + | + | + | - | + |
| Tang QF et al. | 1 | + | + | + | - | + | + | + | - | + |
| Wang BJ et al. | 2 | + | + | - | - | - | + | + | - | - |
| Dang X | 1 | + | + | + | - | + | + | + | - | + |
| Xi MX | 1 | + | + | + | - | + | + | + | - | + |
| Cheng YM | 1 | + | + | + | - | + | + | + | - | + |
| Yin YY | 2 | + | + | + | - | + | + | + | - | + |
| Zheng XM | 3 | + | + | + | - | + | + | + | - | + |
| Bu F et al. | 1 | - | - | - | - | + | + | + | - | + |
| Deng YH et al. | 1 | - | + | + | - | + | + | + | - | + |
| Dong XT et al. | 1 | + | + | + | - | - | + | + | - | - |
| Wang XW and Xu YL | 1 | + | + | + | - | + | + | + | - | + |
| Wang ZJ et al. | 1 | + | + | + | - | + | + | + | - | + |
| Liu Y | 1 | + | + | + | - | + | + | + | - | + |
| Xiao RF | 1 | - | + | + | - | + | + | + | - | + |
| Wu JQ | 5 | + | + | - | - | + | + | + | - | + |
| Meng L | 1 | + | + | + | - | + | + | + | - | + |

^a^ROB, risk of bias;

^b^+indicates low ROB/low concern regarding applicability;

^c^? indicates unclear ROB/unclear concern regarding applicability

^d^-indicates high ROB/high concern regarding application.

**Table S5.** Performance metrics (true-positive, false-positive, true-negative and false-negative values) of 17 studies to identify frailty and cognitive frailty in elderly patients with diabetes

| Author | N | Frailty/Cognitive frailty | TP | FP | FN | TN |
| --- | --- | --- | --- | --- | --- | --- |
| Wu JX et al. | 509 | 148 | 64 | 5 | 84 | 356 |
| Xiao RF et al. | 1107 | 113 | 85 | 203 | 28 | 791 |
| Ma SM et al. | 253 | 76 | 66 | 39 | 10 | 138 |
| Wang SJ et al. | 202 | 80 | 56 | 12 | 24 | 110 |
| Liang MY et al. | 265 | 93 | 80 | 39 | 13 | 133 |
| Liu XX and Fang XZ | 220 | 137 | 127 | 11 | 10 | 72 |
| Wang BJ et al. | 491 | 216 | 202 | 20 | 14 | 255 |
| Dang X | 360 | 115 | 94 | 32 | 21 | 213 |
| Xi MX | 338 | 130 | 114 | 61 | 16 | 147 |
| Cheng YM | 317 | 118 | 109 | 19 | 9 | 180 |
| Yin YY | 379 | 194 | 145 | 25 | 49 | 160 |
| Zheng XM | 380 | 112 | 82 | 40 | 30 | 228 |
| Bu F et al. | 1436 | 145 | 119 | 263 | 26 | 1028 |
| Dong XT et al. | 485 | 211 | 164 | 71 | 47 | 203 |
| Liu Y | 483 | 98 | 69 | 54 | 29 | 331 |
| Meng L | 508 | 117 | 93 | 93 | 24 | 298 |
| Wang ZJ et al. | 321 | 85 | 56 | 34 | 29 | 202 |

# Figures

**Figure S1.** Forest plot of pooled area under the receiver operating characteristic curve (AUC) estimates from the meta-analysis of 32 models for frailty and cognitive frailty in elderly patients with diabetes. AUC:area under the curve; LR:logistic regression; RF: random forest; SVM: support vector machine; KNN: k-nearest neighbors; DT: decision tree; NN: neural network.

**
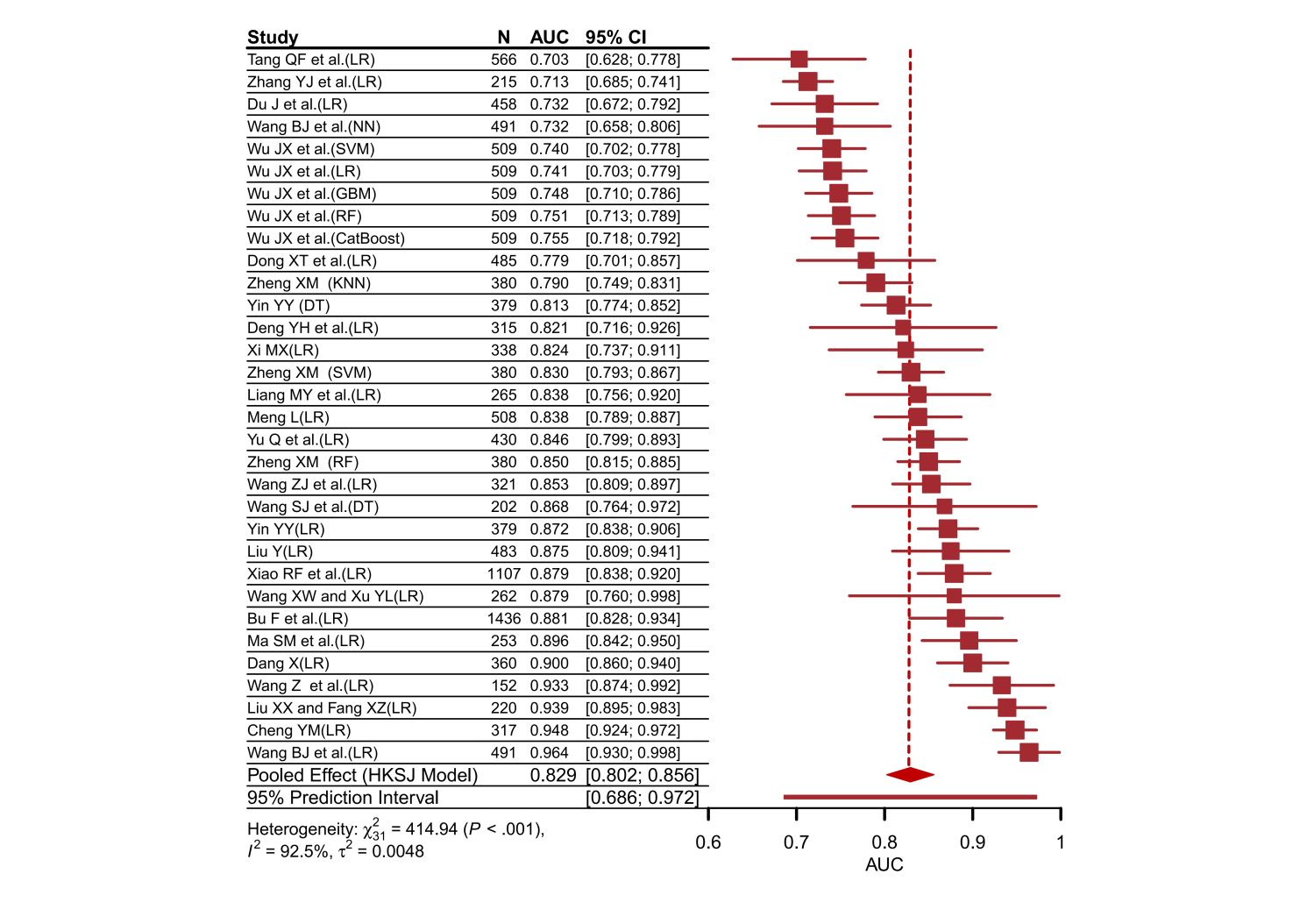
**

**Figure S2.** Deeks' funnel plot asymmetry test forpublication bias among the 17 included studies.


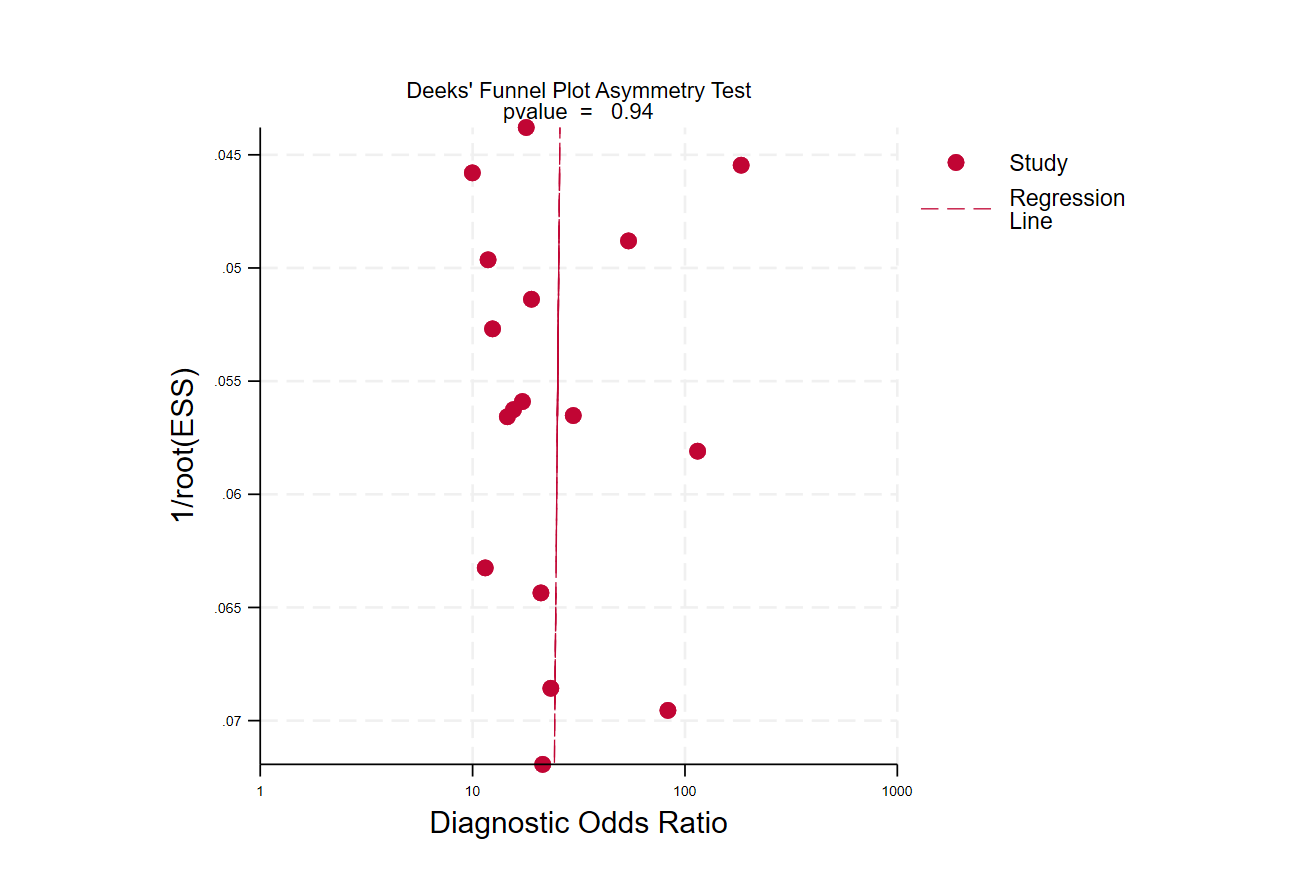

Supplement: Multimedia Appendix 1 [file jmir-v28-e84617-s001.docx]
