# Supplementary material for: Characterization of Models for Identifying Physical and Cognitive Frailty in Older Adults With Diabetes: Systematic Review and Meta-Analysis
Source: J Med Internet Res. 2026 Jan 29;28:e84617. doi: 10.2196/84617 (PMC12854664; doi:10.2196/84617)
Supplement: Checklist 1 [file jmir-v28-e84617-s002.pdf]

PRISMA 2020 expanded checklist

Section and Topic	Item #	Checklist Item	Reported on page #
TITLE			
Title	1	Identify the report as a systematic review.	1
ABSTRACT			
Abstract	2	See the PRISMA 2020 for Abstracts checklist.	2
INTRODUCTION			
Rationale	3	Describe the rationale for the review in the context of existing knowledge.	3-4
Objectives	4	Provide an explicit statement of the objective(s) or question(s) the review addresses.	4
METHODS			
Eligibility criteria	5	Specify the inclusion and exclusion criteria for the review and how studies were grouped for the syntheses.	5
Information sources	6	Specify all databases, registers, websites, organisations, reference lists and other sources searched or consulted to identify studies. Specify the date when each source was last searched or consulted.	4-5
Search strategy	7	Present the full search strategies for all databases, registers and websites, including any filters and limits used.	4-5
Selection process	8	Specify the methods used to decide whether a study met the inclusion criteria of the review, including how many reviewers screened each record and each report retrieved, whether they worked independently, and if applicable, details of automation tools used in the process.	5
Data collection process	9	Specify the methods used to collect data from reports, including how many reviewers collected data from each report, whether they worked independently, any processes for obtaining or confirming data from study investigators, and if applicable, details of automation tools used in the process.	5-6
Data items (outcomes)	10a	List and define all outcomes for which data were sought. Specify whether all results that were compatible with each outcome	5

		domain in each study were sought (e.g. for all measures, time points, analyses), and if not, the methods used to decide which results to collect.	
Data items (other variables)	10b	List and define all other variables for which data were sought (e.g. participant and intervention characteristics, funding sources). Describe any assumptions made about any missing or unclear information.	5
Study risk of bias assessment	11	Specify the methods used to assess risk of bias in the included studies, including details of the tool(s) used, how many reviewers assessed each study and whether they worked independently, and if applicable, details of automation tools used in the process.	6
Effect measures	12	Specify for each outcome the effect measure(s) (e.g. risk ratio, mean difference) used in the synthesis or presentation of results.	6
Synthesis methods (eligibility for synthesis)	13a	Describe the processes used to decide which studies were eligible for each synthesis (e.g. tabulating the study intervention characteristics and comparing against the planned groups for each synthesis (item #5)).	6
Synthesis methods (preparing for synthesis)	13b	Describe any methods required to prepare the data for presentation or synthesis, such as handling of missing summary statistics, or data conversions.	6
Synthesis methods (tabulation and graphical methods)	13c	Describe any methods used to tabulate or visually display results of individual studies and syntheses.	7
Synthesis methods (statistical synthesis methods)	13d	Describe any methods used to synthesize results and provide a rationale for the choice(s). If meta-analysis was performed, describe the model(s), method(s) to identify the presence and extent of statistical heterogeneity, and software package(s) used.	6-7
Synthesis methods (methods to explore heterogeneity)	13e	Describe any methods used to explore possible causes of heterogeneity among study results (e.g. subgroup analysis, meta-regression).	6-7
Synthesis methods (sensitivity)	13f	Describe any sensitivity analyses conducted to assess robustness of the synthesized results.	7
Reporting bias assessment	14	Describe any methods used to assess risk of bias due to missing results in a synthesis (arising from reporting biases).	7
Certainty assessment	15	Describe any methods used to assess certainty (or confidence) in the body of evidence for an outcome.	6
RESULTS			

Study selection(flow of studies)	16a	Describe the results of the search and selection process, from the number of records identified in the search to the number of studies included in the review, ideally using a flow diagram.	7
Study selection (excluded)	16b	Cite studies that might appear to meet the inclusion criteria, but which were excluded, and explain why they were excluded.	7
Study characteristics	17	Cite each included study and present its characteristics.	7-8, 23, 27
Risk of bias in studies	18	Present assessments of risk of bias for each included study.	9
Results of individual studies	19	For all outcomes, present, for each study: (a) summary statistics for each group (where appropriate) and (b) an effect estimate and its precision (e.g. confidence/credible interval), ideally using structured tables or plots.	23-26
Results of syntheses (characteristics)	20a	For each synthesis, briefly summarise the characteristics and risk of bias among contributing studies.	7-9
Results of syntheses (statistical)	20b	Present results of all statistical syntheses conducted. If meta-analysis was done, present for each the summary estimate and its precision (e.g. confidence/credible interval) and measures of statistical heterogeneity. If comparing groups, describe the direction of the effect.	10-11
Results of syntheses (heterogeneity)	20c	Present results of all investigations of possible causes of heterogeneity among study results.	11
Results of syntheses (sensitivity)	20d	Present results of all sensitivity analyses conducted to assess the robustness of the synthesized results.	10-11
Reporting biases	21	Present assessments of risk of bias due to missing results (arising from reporting biases) for each synthesis assessed.	11
Certainty of evidence	22	Present assessments of certainty (or confidence) in the body of evidence for each outcome assessed.	14-16
DISCUSSION			
Interpretation	23a	Provide a general interpretation of the results in the context of other evidence.	11-13
Limitations of evidence	23b	Discuss any limitations of the evidence included in the review.	14-15
Limitations of review processes	23c	Discuss any limitations of the review processes used.	16
Implications	23d	Discuss implications of the results for practice, policy, and future research.	16-17
OTHER			

INFORMATION			
Registration and protocol (registration)	24a	Provide registration information for the review, including register name and registration number, or state that the review was not registered.	3, 4
Registration and protocol (protocol)	24b	Indicate where the review protocol can be accessed, or state that a protocol was not prepared.	3
Registration and protocol(amendments)	24c	Describe and explain any amendments to information provided at registration or in the protocol.	3
Support	25	Describe sources of financial or non-financial support for the review, and the role of the funders or sponsors in the review.	3, 18
Competing interests	26	Declare any competing interests of review authors.	17
Availability of data, code, and other materials	27	Report which of the following are publicly available and where they can be found: template data collection forms; data extracted from included studies; data used for all analyses; analytic code; any other materials used in the review.	17

PRISMA-DTA Checklist

Section/topic	#	PRISMA-DTA Checklist Item	Reported on page #
TITLE / ABSTRACT			
Title	1	Identify the report as a systematic review (+/- meta-analysis) of diagnostic test accuracy (DTA) studies.	1
Abstract	2	Abstract: See PRISMA-DTA for abstracts.	2
INTRODUCTION			
Rationale	3	Describe the rationale for the review in the context of what is already known.	3-4
Clinical role of index test	D1	State the scientific and clinical background, including the intended use and clinical role of the index test, and if applicable, the rationale for minimally acceptable test accuracy (or minimum difference in accuracy for comparative design).	3

Objectives	4	Provide an explicit statement of question(s) being addressed in terms of participants, index test(s), and target condition(s).	4
METHODS			
Protocol and registration	5	Indicate if a review protocol exists, if and where it can be accessed (e.g., Web address), and, if available, provide registration information including registration number.	3, 4
Eligibility criteria	6	Specify study characteristics (participants, setting, index test(s), reference standard(s), target condition(s), and study design) and report characteristics (e.g., years considered, language, publication status) used as criteria for eligibility, giving rationale.	5
Information sources	7	Describe all information sources (e.g., databases with dates of coverage, contact with study authors to identify additional studies) in the search and date last searched.	4-5
Search	8	Present full search strategies for all electronic databases and other sources searched, including any limits used, such that they could be repeated.	5
Study selection	9	State the process for selecting studies (i.e., screening, eligibility, included in systematic review, and, if applicable, included in the meta-analysis).	5
Data collection process	10	Describe method of data extraction from reports (e.g., piloted forms, independently, in duplicate) and any processes for obtaining and confirming data from investigators.	5-6
Definitions for data extraction	11	Provide definitions used in data extraction and classifications of target condition(s), index test(s), reference standard(s) and other characteristics (e.g. study design, clinical setting).	5, 7
Risk of bias and applicability	12	Describe methods used for assessing risk of bias in individual studies and concerns regarding the applicability to the review question.	6
Diagnostic accuracy measures	13	State the principal diagnostic accuracy measure(s) reported (e.g. sensitivity, specificity) and state the unit of assessment (e.g. per-patient, per-lesion).	5-6
Synthesis of results	14	Describe methods of handling data, combining results of studies and describing variability between studies.	6-7
Meta-analysis	D2	Report the statistical methods used for meta-analyses, if performed.	6-7
Additional analyses	16	Describe methods of additional analyses (e.g., sensitivity or subgroup analyses, meta-regression), if done, indicating which	7

		were pre-specified.	
RESULTS			
Study selection	17	Provide numbers of studies screened, assessed for eligibility, included in the review (and included in meta-analysis, if applicable) with reasons for exclusions at each stage, ideally with a flow diagram.	7
Study characteristics	18	For each included study provide citations and present key characteristics including: a) participant characteristics, b) clinical setting, c) study design, d) target condition definition, e) index test, f) reference standard, g) sample size, h) funding sources.	7-8, 23-28
Risk of bias and applicability	19	Present evaluation of risk of bias and concerns regarding applicability for each study.	9-10
Results of individual studies	20	For each analysis in each study report 2x2 data (TP, FP, FN, TN) with estimates of diagnostic accuracy and confidence intervals, ideally with a forest or ROC plot.	23-26
Synthesis of results	21	Describe test accuracy, including variability; if meta-analysis was done, include results and confidence intervals.	10
Additional analysis	23	Give results of additional analyses, if done (e.g., sensitivity or subgroup analyses, meta-regression).	10-11
DISCUSSION			
Summary of evidence	24	Summarize the main findings including the strength of evidence.	11
Limitations	25	Discuss limitations from included studies (e.g. risk of bias and concerns regarding applicability) and from the review process.	16
Conclusions	26	Provide a general interpretation of the results in the context of other evidence. Discuss implications for future research and clinical practice.	16-17
FUNDING			
Funding	27	For the systematic review, describe the sources of funding and other support and the role of the funders.	3, 18

PRISMA-S Checklist

Section/topic	#	Checklist item	Locatio n(s)

			Reporte d
INFORMATION SOURCES AND METHODS			
Database name	1	Name each individual database searched, stating the platform for each.	2, 4
Multi-database searching	2	If databases were searched simultaneously on a single platform, state the name of the platform, listing all of the databases searched.	4
Study registries	3	List any study registries searched.	5
Online resources and browsing	4	Describe any online or print source purposefully searched or browsed (e.g., tables of contents, print conference proceedings, web sites), and how this was done.	5
Citation searching	5	Indicate whether cited references or citing references were examined, and describe any methods used for locating cited/citing references (e.g., browsing reference lists, using a citation index, setting up email alerts for references citing included studies).	5
Contacts	6	Indicate whether additional studies or data were sought by contacting authors, experts, manufacturers, or others.	6
Other methods	7	Describe any additional information sources or search methods used.	6-7
SEARCH STRATEGIES			
Full search strategies	8	Include the search strategies for each database and information source, copied and pasted exactly as run.	5
Limits and restrictions	9	Specify that no limits were used, or describe any limits or restrictions applied to a search (e.g., date or time period, language, study design) and provide justification for their use.	2, 4, 5
Search filters	10	Indicate whether published search filters were used (as originally designed or modified), and if so, cite the filter(s) used.	4-5
Prior work	11	Indicate when search strategies from other literature reviews were adapted or reused for a substantive part or all of the	4-5

		search, citing the previous review(s).	
Updates	12	Report the methods used to update the search(es) (e.g., rerunning searches, email alerts).	4-5
DATES			
Dates of searches	13	For each search strategy, provide the date when the last search occurred.	2, 4
PEER REVIEW			
Peer review	14	Describe any search peer review process.	5
MANAGING RECORDS			
Total Records	15	Document the total number of records identified from each database and other information sources.	7
Deduplication	16	Describe the processes and any software used to deduplicate records from multiple database searches and other information sources.	5
